# Serum retinol-binding protein 4 is associated with the presence and severity of coronary artery disease in patients with subclinical hypothyroidism

**DOI:** 10.18632/aging.102065

**Published:** 2019-07-06

**Authors:** Hui-Xian Sun, Hui-Hong Ji, Xiao-Lin Chen, Li Wang, Yue Wang, Xi-Yu Shen, Xiang Lu, Wei Gao, Lian-Sheng Wang

**Affiliations:** 1 Department of Geriatrics, Sir Run Run Hospital, Nanjing Medical University, Nanjing, China; 2 Key Laboratory for Aging and Disease, Nanjing Medical University, Nanjing, China; 3 Department of Cardiology, The First Affiliated Hospital of Nanjing Medical University, Nanjing, China; 4 Department of Internal Medicine, The Hospital of HoHai University, Nanjing, China; 5 Department of Respiratory, Sir Run Run Hospital, Nanjing Medical University, Nanjing, China

**Keywords:** retinol-binding protein 4, coronary artery disease, subclinical hypothyroidism

## Abstract

Subclinical hypothyroidism (SCH) plays a crucial role in the development and progression of coronary heart disease (CAD). Retinol-binding protein 4 (RBP4) is an adipokine correlated with cardiovascular diseases. Recent studies found that RBP4 levels are increased in patients with SCH. However, the relationship of RBP4 with CAD in SCH patients remains unclear. A total of 199 SCH patients (148 with CAD and 51 without CAD) and 102 healthy controls were enrolled in this study. Serum RBP4 was increased in SCH patients than controls. Moreover, serum RBP4 was higher in SCH patients with CAD. Although there was no significant difference of metabolic parameters between SCH patients with and without CAD, serum RBP4 was positively correlated with body mass index, total cholesterol, and low-density lipoprotein cholesterol, as well as thyroid stimulating hormone. Multivariable logistic regression analyses revealed elevated RBP4 was correlated with increased risk for CAD in SCH patients. Serum RBP4 levels were also increased as the number of stenosed vessels increased. Furthermore, increased RBP4 was positively correlated with the severity of CAD quantified by the Gensini score. Our findings demonstrate that serum RBP4 is associated with the presence and severity of CAD in patients with SCH.

## INTRODUCTION

Coronary artery disease (CAD) remains the leading cause of morbidity and mortality worldwide, especially in the elderly population [1]. Despite of several common risk factors, including smoking, alcohol, hyperlipidemia, and obesity *etc.* [2], several classical hormones also play vital roles in the development of CAD. It is generally accepted that thyroid hormone (TH) has anti-atherosclerotic effects and hypothyroidism accelerates the process of atherogenesis [3]. Epidemiological studies have suggested an increased risk and severity of CAD in patients with hypothyroidism [4]. Subclinical hypothyroidism (SCH) is an early, mild form of hypothyroidism with a state of increased thyroid stimulating hormone (TSH) levels but normal total or free thyroxine (T4) levels [5]. SCH is a common health problem, with a prevalence of about 10% in the population without known thyroid disease [5]. The prevalence of SCH increased with age and ranged from 7% to 26% in the elderly [6]. The Rotterdam study showed that patients with SCH had higher susceptibility of myocardial infarction than those with euthyroid [7], indicating the importance of risk factor screening for CAD in SCH pateints. However, due to lack of explicit clinical signs and symptoms, novel and convenient markers are needed for the prediction of CAD in patients with SCH.

Retinol-binding protein 4 (RBP4) is an approximately 21-kDa secreted protein that transports retinol (vitamin A) in circulation [8]. RBP4 has been well known as an important adipokine that contributes to insulin resistance both in rodent and human [9, 10]. Recent clinical studies have also linked higher circulating RBP4 to cardiovascular diseases, including hypertension [11, 12], heart failure [13, 14], and atherosclerosis [15–19]. Remarkably, the association of elevated plasma RBP4 level with increased risk of CAD was confirmed among women in the Nurses' Health Study [20]. Although most of the following studies [21–24] observed similar results showing that increased RBP4 level was correlated with CAD, a prospective study demonstrated no significant relationship between serum RBP4 and the risk of CAD [25]. The discrepancies among different studies might attribute to the heterogeneity in study design, age, race, and other participant characteristics. Our recent study also showed that RBP4 levels were decreased in men with CAD but not changed in women with CAD, indicating that sex hormone levels may affect the role of RBP4 in the development of CAD [26]. Interestingly, clinical studies showed that RBP4 were increased in patients with SCH and were positively correlated to the level of TSH [27–29]. However, the effect of RBP4 in predicting CAD in patients with SCH remains unclear. Considering SCH is a well-known risk factor for coronary atherosclerosis, we carried out the present study to evaluate the association of serum RBP4 concentration with the presence and severity of angiographically demonstrated CAD in patients with SCH.

## RESULTS

### Characteristics of the study populations

The anthropometric and biochemical characteristics are presented in Table 1. No statistically significant differences were observed between SCH patients and control subjects regarding age, sex, BMI, smoking, hypertension, diabetes, blood pressure, and the levels of TC, TG, LDL-C, ApoA, FBG, ALT, AST, Cr, and UA. When compared with control subjects, SCH patients had higher levels of TSH and RBP4 (*P* < 0.01), but lower FT4 (*P* = 0.005).

**Table 1:** Characteristics of the participants.

**Variables**	**Controls (n = 102)**	**SCH (n = 199)**	***P* value**	**SCH (n = 199)**		***P* value**
				**Without CAD (n = 51)**	**With CAD (n = 148)**	
Age (years)	63.8 ± 8.2	64.4 ± 10.9	0.644	62.7 ± 10.7	65.0 ± 11.0	0.202
Male, n (%)	54 (52.9)	92 (46.2)	0.270	21 (41.2)	71 (48.0)	0.401
BMI (kg/m^2^)	24.3 (23.4–25.4)	25.0 (23.0–27.0)	0.250	25.6 (23.0–28.4)	24.8 (23.0–27.0)	0.137
Smoking, n (%)	47 (46.1)	83 (41.7)	0.469	18 (35.3)	65(43.9)	0.281
Hypertension, n (%)	74 (72.5)	127 (63.8)	0.128	33 (64.7)	94 (63.5)	0.879
Diabetes, n (%)	25 (24.5)	67 (33.7)	0.103	21 (41.2)	46 (31.1)	0.188
SBP, mmHg	135.0 (125.0–145.0)	132.0 (120.0–145.0)	0.374	130.0 (120.0–150.0)	135.0 (120.0–145.0)	0.689
DBP, mmHg	80.0 (70.0–86.0)	80.0 (70.0–85.0)	0.874	78.0 (70.0–90.0)	80.0 (71.3–95.0)	0.251
TC (mmol/L)	4.07 (3.63–4.53)	3.98 (3.41–4.94)	0.582	3.74 (3.34–4.48)	4.20 (3.47–4.98)	0.098
TG (mmol/L)	1.23 (0.98–1.58)	1.34 (0.99–1.94)	0.100	1.41 (1.05–2.25)	1.31 (0.99–1.86)	0.525
LDL-C (mmol/L)	2.54 ± 0.79	2.74 ± 0.88	0.066	2.55 ± 0.87	2.80 ± 0.88	0.074
HDL-C (mmol/L)	1.13 ± 0.28	1.04 ± 0.24	0.002	1.09 ± 0.29	1.02 ± 0.22	0.058
ApoA (nmol/L)	150.0 (116.0–265.0)	189.0 (113.0–331.0)	0.341	132.0 (61.0–339.0)	202.5 (124.5–325.0)	0.037
FBG (mg/dL)	97.92 (90.36–109.44)	96.12 (87.12–114.30)	0.689	107.46 (95.94–126.36)	113.76 (97.74–144.36)	0.137
ALT (U/L)	24.4 (23.4–25.8)	24.8 (22.6–27.6)	.0338	25.3 (23.7–27.4)	24.5 (22.3–27.7)	0.174
AST (U/L)	25.3 (23.9–26.9)	23.2 (19.3–32.7)	0.051	23.2 (19.3–32.6)	23.4 (19.3–32.9)	0.869
Cr (μmol/L)	77.32 (73.51–83.55)	75.80 (65.90–87.70)	0.223	77.26 (72.53–83.99)	77.64 (72.53–85.66)	0.235
UA (μmol/L)	341.73 ± 94.47	341.91 ± 97.57	0.732	339.24 ± 94.88	342.83 ± 98.78	0.821
TSH (mIU/L)	3.45 (3.03–3.71)	4.30 (4.23–4.40)	< 0.001	4.25 (4.22–4.36)	4.32 (4.24–4.41)	0.038
FT3 (pmol/L)	4.32 ± 0.43	4.28 ± 0.47	0.685	4.36 ± 0.50	4.26 ± 0.46	0.190
T3 (nmol/L)	1.93 ± 0.23	1.94 ± 0.44	0.923	2.05 ± 0.46	1.90 ± 0.44	0.037
FT4 (pmol/L)	16.00 (13.40–19.60)	15.10 (14.70–15.70)	0.010	15.10 (14.80–15.70)	15.10 (14.60–15.70)	0.345
T4 (nmol/L)	126.15 ± 9.82	122.58 ± 10.31	0.653	123.86 ± 9.56	122.20 ± 11.50	0.356
RBP4 (μg/mL)	35.07 ± 10.44	54.84 ± 12.30	< 0.001	42.58 ± 8.76	58.29 ± 13.86	< 0.001

Data are mean ± standard deviation, median with interquartile range in parenthesis, or number with percentage in parenthesis.

CAD, coronary artery disease; SCH, subclinical hypothyroidism; BMI, body mass index; SBP, systolic blood pressure; DBP, diastolic blood pressure; TC, total cholesterol; TG, triglyceride; HDL-C, high-density lipoprotein cholesterol; LDL-C, low-density lipoprotein cholesterol; ApoA, Apolipoprotein A; FBG, fasting blood glucose; ALT, alanine aminotransferase; AST, aspartate aminotransferase; Cr, creatine; UA, uric acid; TSH, thyroid stimulating hormone; FT3, free triiodothyronine; T3, Triiodothyronine; FT4, free thyroxine; T4, thyroxine; RBP4, retinol-binding protein 4.

SCH patients were divided into 148 patients with CAD (mean age 65.0 ± 11.0 years) and 51 patients without CAD (mean age 62.7 ± 10.7 years). When compared to those without CAD, patietns with CAD had higher ApoA (median: 202.5 nmol/L *vs.* 132.0 nmol/L, *P* = 0.037) and TSH (median: 4.32 mIU/L *vs.* 4.25mIU/L, *P* = 0.038), but lower T3 levels (mean: 1.90 nmol/L *vs.* 2.05 nmol/L, *P* = 0.037). Moreover, the RBP4 concentrations were also increased in patients with CAD (mean: 58.29 μg/mL vs. 42.58 μg/mL, *P* < 0.001). No statistically significant differences were found between these two groups regarding age, sex, BMI, smoking, hypertension, diabetes, blood pressure, and the levels of TC, TG, HDL-C, LDL-C, FBG, ALT, AST, Cr, UA, FT3, FT4, and T4.

### Correlation of serum RBP4 with clinical parameters in patients with SCH

**Figure 1:**
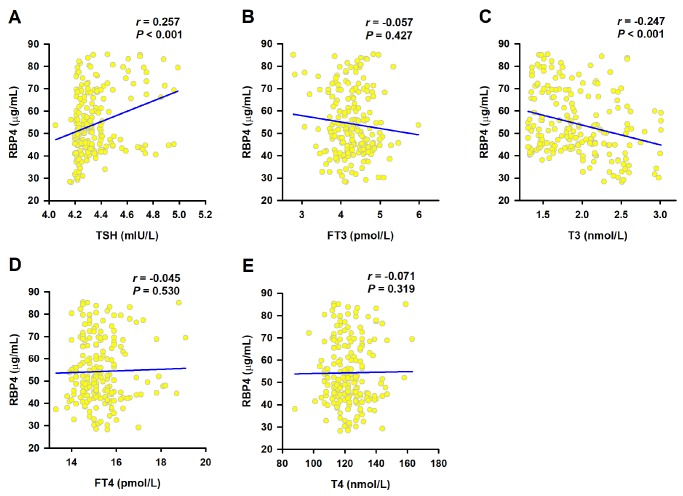
**Correlation of serum RBP4 with thyroid function.** Spearman correlation coefficient was used to analyze the association of serum RBP4 levels with TSH (**A**), FT3 (**B**), T3 (**C**), FT4 (**D**) and T4 (**E**).

We next evaluated the association of RBP4 with clinical parameters in SCH patients (Table 2). In CAD group, the levels of RBP4 were positively correlated with TC (*r* = 0.250, *P* = 0.002), LDL-C (*r* = 0.276, *P* = 0.001), and BMI (*r* = 0.168, *P* = 0.042). Serum RBP4 were also positively related to BMI (*r* = 0.357, *P* = 0.010) in patients without CAD. We then analyzed the association of serum RBP4 with thyroid function. As shown in Figure 1, RBP4 concentrations were positively associated with TSH (*r* = 0.257, *P* < 0.001), but negatively correlated with T3 (*r* = -0.247, *P* < 0.001). The correlation of RBP4 with TSH (*r* = 0.241; *P* = 0.004) and T3 (r = -0.158; *P* = 0.018) remained significant even after adjustments for potential confounders including age, sex, smoking, hypertension, diabetes, BMI, and the levels of Cr, FBG, TC, TG, HDL-C, and LDL-C.

**Table 2:** Spearman’s correlation of serum RBP4 with clinical parameters in patients with SCH.

**Variables**	**Without CAD (n = 51)**	***P* value**	**With CAD (n = 148)**	***P* value**
Age	0.112	0.433	0.125	0.131
BMI	0.357	0.010	0.168	0.042
SBP	0.019	0.896	0.089	0.282
DBP	0.012	0.933	0.018	0.829
TC	0.039	0.788	0.250	0.002
TG	0.022	0.878	0.079	0.342
LDL-C	0.079	0.582	0.276	0.001
HDL-C	−0.095	0.506	0.014	0.863
ApoA	0.001	0.999	−0.048	0.561
FBG	0.017	0.903	0.143	0.084
ALT	0.227	0.109	−0.054	0.517
AST	0.144	0.314	−0.077	0.349
Cr	0.061	0.671	0.085	0.305
UA	0.045	0.753	0.094	0.257

SCH, subclinical hypothyroidism; CAD, coronary artery disease; BMI, body mass index; SBP, systolic blood pressure; DBP, diastolic blood pressure; TC, total cholesterol; TG, triglyceride; HDL-C, high-density lipoprotein cholesterol; LDL-C, low-density lipoprotein cholesterol; ApoA, Apolipoprotein A; FBG, fasting blood glucose; ALT, alanine aminotransferase; AST, aspartate aminotransferase; Cr, creatine; UA, uric acid; RBP4, retinol-binding protein 4.

### Correlation of serum RBP4 with the presence of CAD in patients with SCH

**Figure 2:**
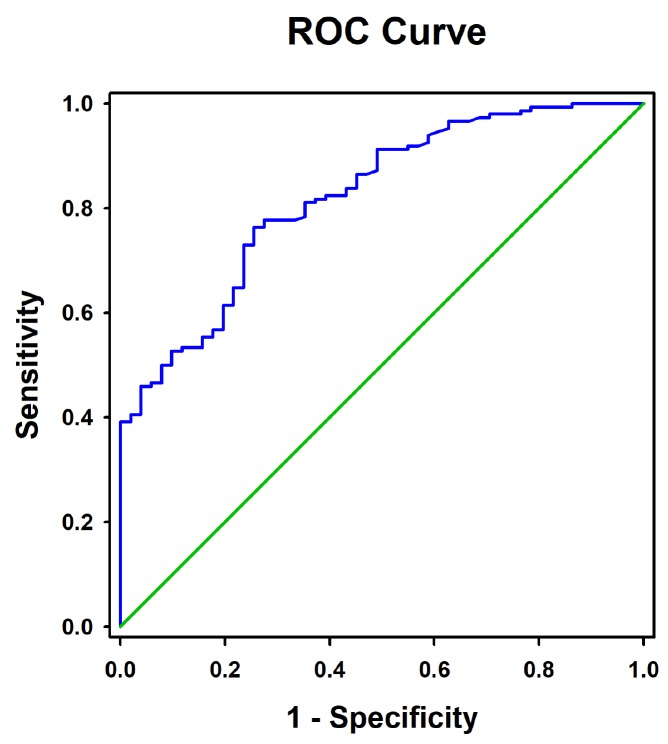
**Receiver operating characteristic curves for the diagnostic accuracy of RBP4 for CAD.**

As shown in Figure 2, ROC curve analysis showed that the optimal cut-off value of RBP4 for the prediction of CAD was 45.90 μg/mL, with a sensitivity of 76.4% and a specificity of 74.5% (area under the curve = 0.822, 95%CI = 0.76 – 0.88, *P* < 0.0001). Univariate and multivariate logistic regression demonstrated that elevated RBP4 concentration was associated with the presence of CAD even after adjustment for the above mentioned potential confounders (*P* < 0.001) (Table 3). Similar results were obtained by using RBP4 as a continuous variable (adjusted OR = 1.17, 95%CI = 1.10 – 1.25, *P* = 0.001).

**Table 3:** Associations of serum RBP4 with the presence of CAD in patients with SCH.

	**Categorical**		**Continuous**	
	**OR (95%CI)**	***P* value**	**OR (95%CI)**	***P* value**
Crude model	1.39 (1.14–1.89)	< 0.001	1.14 (1.09–1.19)	0.001
Adjusted model	1.71 (1.16–2.46)	< 0.001	1.17 (1.10–1.25)	0.001

The adjusted model included age, gender, BMI, smoking, hypertension, diabetes, TSH, T3, TC, TG, LDL-C, HDL-C, FBG, UA, and Cr.

SCH, subclinical hypothyroidism; CAD, coronary artery disease; OR, odds ratio; CI, confidence interval.

### Correlation of serum RBP4 level with the severity of CAD in patients with SCH

**Figure 3:**
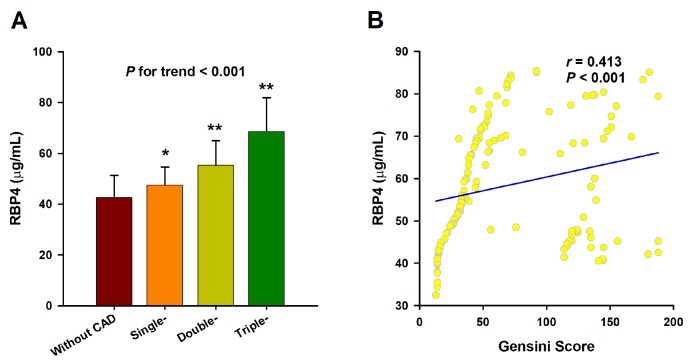
**Association of RBP4 with the severity of CAD.** (**A**) Serum RBP4 level increases as the number of affected vessels increases. The analysis was adjusted for age, sex, BMI, smoking, hypertension, diabetes, TC, TG, LDL-C, HDL-C, ApoA, TSH, T3, FBG, UA and Cr. The *P* value for test for trend of the changes of serum RBP4 concentrations across the severity of coronary angiography is given. *, *P* < 0.01 vs. Control; **, *P* < 0.001 vs. Control. (**B**) Spearman correlation coefficient was used to analyze the correlation between serum RBP4 levels and Gensini scores.

CAD patients were divided into single-, double-, and triple-vessel disease subgroups according to the number of significantly stenosed vessels. As the number of stenosed vessels increased, serum levels of RBP4 significantly increased (mean: 20.0 μg/mL vs. 25.0 μg/mL vs. 34.5 μg/mL, *P* = 0.015). Linear regression analysis revealed that RBP4 concentrations were also increased as the stenosed vessels increased (*β* = 0.247, *P* for trend < 0.001) (Figure 3A). The trends of RBP4 concentrations across the severity of CAD remained significant (*β* = 0.203, *P* for trend < 0.001) even after adjustment for the aforementioned confounders. Moreover, serum RBP4 levels were positively correlated with the Gensini score (*r* = 0.413, *P* < 0.001), which is also an indicator for the severity of CAD (Figure 3B).

### Stratification analyses for the association of Serum RBP4 with the presence and severity of CAD in patients with SCH

Stratified analyses were further conducted according to diabetes and sex status (Table 4). We first divided the SCH patients into diabetes (n = 67) and non-diabetes (n = 132) subgroups. There was no significant difference of RBP4 levels between these two groups (mean: 55.79 μg/mL *vs.* 54.35 μg/mL, *P* = 0.621). After exclusion of patients with diabetes, patients with elevated RBP4 concentrations were correlated with higher risk of CAD even after adjustment for the aforementioned confounders (adjusted OR = 1.82, 95%CI = 1.46 – 2.13, *P* < 0.001). Similar results were obtained by using serum RBP4 as a continuous variable (adjusted OR = 1.17, 95%CI = 1.07 – 1.26, *P* < 0.001). In addition, there was also no significant difference of serum RBP4 between men and women (mean: 54.79 μg/mL *vs.* 54.88 μg/mL, *P* = 0.974). Elevated serum RBP4 levels were correlated with higher risks of CAD both in male and female patients.

**Table 4:** Stratification analyses for the association of serum RBP4 with the presence of CAD in patients with SCH.

	**Categorical**				**Continuous**			
	**Crude OR (95%CI)**	***P* value**	**Adjusted OR (95%CI)**	***P* value**	**Crude OR (95%CI)**	***P* value**	**Adjusted OR (95%CI)**	***P* value**
Diabetes								
With diabetes	1.11 (1.03 – 1.17)	<0.001	1.41 ^1^ (0.78 – 2.17)	0.066	1.15 (1.07 – 1.23)	<0.001	1.27 ^1^ (0.99 – 1.61)	0.052
Without diabetes	1.57 (1.39 – 1.86)	<0.001	1.82 ^1^ (1.46 – 2.13)	<0.001	1.15 (1.08 – 1.22)	<0.001	1.17 ^1^ (1.07 – 1.27)	<0.001
Gender								
Male	1.16 (1.03 – 1.19)	<0.001	1.61 ^2^ (1.29 – 1.92)	0.004	1.20 (1.09 – 1.31)	<0.001	1.26 ^2^ (1.09 – 1.48)	0.002
Female	1.89 (1.34 – 2.31)	<0.001	1.64 ^2^ (1.17 – 1.85)	<0.001	1.12 (1.06 – 1.17)	<0.001	1.18 ^2^ (1.09 – 1.28)	<0.001

^1^ The adjusted model included age, gender, BMI, smoking, hypertension, TSH, T3, TC, TG, LDL-C, HDL-C, FBG, UA, and Cr.

^2^ The adjusted model included age, BMI, smoking, hypertension, diabetes, TSH, T3, TC, TG, LDL-C, HDL-C, FBG, UA, and Cr.

SCH, subclinical hypothyroidism; CAD, coronary artery disease; OR, odds ratio; CI, confidence interval.

## DISCUSSION

Our present study demonstrated that serum RBP4, a well-known adipokine with adverse effects on cardiovascular system, was not only correlated with thyroid dysfunction, but also strongly associated with CAD in patients with SCH. Among 199 patients with SCH in our study, subjects with elevated serum RBP4 levels were correlated with nearly 1.7-fold increase in the risk of CAD. Furthermore, we also found that RBP4 concentration was positively correlated with the severity of CAD.

RBP4 has been shown to correlate with insulin resistance, and its circulating level elevates in diabetes, obesity, and metabolic disorders [30]. Thyroid dysfunction, prominently SCH has been observed more frequently in metabolic syndrome patients than general population [31]. However, the data on the association of RBP4 with thyroid dysfunction are very limited. Now we demonstrated that serum RBP4 concentrations were increased and positively related to TSH concentrations in patients with SCH. These results are consistent with previous studies showing that subjects with subclinical and overt hypothyroidism had higher circulating RBP4 levels than those with normal thyroid function [27–29]. However, it remains unclear whether increased RBP4 are a cause or a result of hypothyroidism. In blood circulation, RBP4 binds to transthyretin (TTR) forming a protein complex that prevents glomerular filtration and reduces renal clearance of RBP4 [16]. Lowering TTR could decrease circulating levels of RBP4 by promoting its renal clearance [32]. Kloting N *et al.* [33] reported that serum TTR were elevated in patients with insulin resistance, indicating that increased TTR may contribute to the elevated RBP4 in these subjects. In humans, thyroxine-binding globulin is the main thyroid hormone-binding protein [32]. Therefore, although TTR is also a carrier protein for T4, its level is not related to thyroid functions [32]. In fact, SCH often presents without any overt symptoms for a long time and may affect the serection of various adipokines including RBP4 [34]. Further *in vivo* and *in vitro* studies are needed to delineate the effects and mechanisms of RBP4 in thyroid dysfunction.

Our results demonstrated that RBP4 levels were positively correlated with TC, LDL-C, and BMI, which are all risk factors for CAD. Increasing numbers of clinical studies have shown that increased circulating RBP4 are not only correlated with established cardiovascular risk factors such as obesity and dyslipidemia, but also correlated with the prevalence of atherosclerotic diseases and CAD [16]. We demonstrated that RBP4 concentrations were elevated in SCH patients with CAD and were independently correlated with the presence and severity of CAD in SCH patients. The exact mechanism by which increased circulating RBP4 promotes the development of CAD remains unclear. In endothelial cells, RBP4 could induce inflammation and mitochondrial dysfunction, both of which play key roles in atherogenesis [35, 36].

Moreover, RBP4 could also promote the abnormal proliferation and migration of vascular smooth muscle cells, which is important for the formation of coronary atherosclerosis [37]. A recent study showed that elevated RBP4 facilitated macrophage-derived foam cell formation through activating cholesterol uptake, and thus accelerated atherosclerosis progression [38]. However, additional in-depth investigation is required to elucidate the precise mechanism governing the pathological effect of RBP4 on the progression of coronary atherosclerosis under and status of thyoid dysfunction.

The strength of the study is that the CAD patients and non-CAD patients were identified by coronary angiography. Several limitations of the present study should also be considered. Firstly, selection bias might underpower our results due to the cross-sectional design. Secondly, as our study was performed in Chinese Han population, our findings need to be confirmed in other regions and ethnicities. Thirdly, the relatively small sample size may cause a potential low power of our results.

## METHODS

### Study population

The study population was composed of patients with SCH and healthy control subjects. A total of 199 patients with SCH were recruited from inpatients admitted to the First Affiliated Hospital of Nanjing Medical University. The patients were received coronary angiography because of angina pectoris or other symptoms or signs of cardiovascular disease. The control subjects were selected during the same period in the same hospital from the health examination centre. All subjects included in this study had no history of significant concomitant diseases, including hyperthyroidism, thyroidectomy, previous anti-thyroid therapy, severe hepatic or renal diseases, bleeding disorders, previous thoracic irradiation therapy, autoimmune disease, and malignant diseases. SCH was defined as elevated TSH levels with normal total or free T4 levels. Hypertension was defined as resting systolic blood pressure (SBP) above 140 mmHg and/or diastolic blood pressure (DBP) above 90 mmHg or in the presence of active treatment with antihypertensive agents. Diabetes mellitus was defined as fasting blood glucose (FBG) >7.0 mmol/L or a diagnosis with diet adjustment or anti-diabetic drug therapy. Dyslipidemia was defined according to guideline of the National Cholesterol Education Program (Adult Treatment Panel III). Written informed consent was obtained from each participant and this study was approved by the Ethics Committee of the First Affiliated Hospital of Nanjing Medical University.

### Coronary angiography

Two cardiologists who were unaware of the patients included in this study assessed the angiograms. CAD was defined as luminal diameter narrowing estimated visually at least 50% in any epicardial coronary artery, including the left main coronary artery, left anterior descending, left circumflex, or right coronary artery. CAD patients were divided into single-, double-, and triple-vessel disease subgroups according to the number of significantly stenosed vessels. The severity of CAD was assessed with the Gensini score system based on the degree of luminal narrowing and its geographic importance [39]. The Gensini system scores the narrowing of the coronary artery lumen as follows: 1%-25% narrowing=1, 26%-50% narrowing=2, 51%-75% narrowing=4, 76%–90% narrowing=8, 91%–99% narrowing=16, and complete occlusion=32. The score is then multiplied by a factor that incorporates the importance of the lesion position in the coronary arterial tree as follows: ×5 for the left main coronary artery, ×2.5 for the proximal left anterior descending or left circumflex coronary artery, ×1.5 for the mid-segment of the left anterior descending, ×1 for the distal left anterior descending, right coronary artery or mid-distal left circumflex, and ×0.5 for any other arteries [40].

### Laboratory measurements

Venous blood sample was collected and separated into serum and cellular fractions within 2 h by centrifugation at 3000 g for 10 min. The supernatant (serum) was collected and further centrifuged at 10000 g for 15 min to completely remove the cell debris. The obtained serum was stored at -80 °C before further analysis. Total cholesterol (TC), triglyceride (TG), high-density lipoprotein cholesterol (HDL-C), low-density lipoprotein cholesterol (LDL-C), and Apolipoprotein A (ApoA) levels were measured enzymatically on a chemistry analyzer (Olympus AU5400, Chemical Ltd., Japan). Glucose levels were measured by a glucose oxidase method (Reagent kit, Diagnostic Chemicals Ltd., UK). TSH, free triiodothyronine (FT3), Triiodothyronine (T3), free thyroxine(FT4), T4 were measured by an electrochemiluminescence method (Cobas 8000, Roche, Japan).

### Serum RBP4 measurements

Serum RBP4 levels were assayed in duplicate by using an a sandwich enzyme-linked immunosorbent assay (ELISA) kit (R&D, Minneapolis, MN, USA) according to the manufacturer's protocol. The intra- and inter-assay coefficients of variance were 2.32% and 2.95%, respectively. The analytic sensitivity of the assays was 0.021 ng/mL.

### Statistical analysis

Normality of distribution was assessed using the Kolmogorov-Smirnov test. Data for age, LDL-C, HDL-C, uric acid (UA), FT3, T3, T4 and RBP4 were normally distributed parameters and presented as the mean ± standard deviation, and comparisons were analyzed by Student’s *t* test. Skewed data, including body mass index (BMI), SBP, DBP, TC, TG, ApoA, FBG, alanine aminotransferase (ALT), aspartate aminotransferase (AST), creatine (Cr), TSH, FT4 and Gensini score were expressed as median and quartile ranges, and comparisons were analyzed by the Mann-Whitney *U* test. Pearson *χ^2^* test was used to compare qualitative variables represented as frequencies. The correlations between serum RBP4 level and other variables were calculated using Spearman correlation coefficient and partial correlation coefficient adjusted for age, sex, smoking, alcohol intake, BMI, Cr, FBG, TC, TG, and LDL-C, as appropriate. Univariate analysis and multivariate logistic regression analysis were taken to determine the variables that independently contributed to the presence of CAD. Odds ratios (ORs) and 95% confidence intervals (CIs) were calculated. Linear regression analysis was used to test for trend of the changes of serum RBP4 concentrations across the severity of coronary angiography (normal to triple-vessel disease). Receiver operating characteristic (ROC) curve analysis was used to determine the optimum cut-off level of RBP4 best predicting CAD. All tests were two-sided and *P* < 0.05 was considered statistically significant. Statistical analyses were performed using PASW 18.0 (IBM SPSS, Inc., Chicago, USA).

## CONCLUSIONS

In summary, our findings support the point that serum RBP4 is associated with the presence and severity of CAD in SCH patients. Further prospective and experimental studies are needed to delineate whether increased RBP4 may participate in the development of coronary atherosclerosis in hypothyroidism.

### Ethics approval

Written informed consent was obtained from each participant and this study was approved by the Ethics Committee of the First Affiliated Hospital of Nanjing Medical University.
